# A High Triglyceride-Glucose Index Is Associated With Contrast-Induced Acute Kidney Injury in Chinese Patients With Type 2 Diabetes Mellitus

**DOI:** 10.3389/fendo.2020.522883

**Published:** 2021-01-22

**Authors:** Yuhan Qin, Haixia Tang, Gaoliang Yan, Dong Wang, Yong Qiao, Erfei Luo, Jiantong Hou, Chengchun Tang

**Affiliations:** ^1^ Department of Cardiology, Medical School of Southeast University, Nanjing, China; ^2^ Department of Cardiology, Zhongda Hospital affiliated with Southeast University, Nanjing, China

**Keywords:** triglyceride-glucose index, insulin resistance, contrast induced-acute kidney injury, type 2 diabetes mellitus, coronary angiology

## Abstract

**Background and Objectives:**

Triglyceride-glucose (TyG) is an emerging vital indicator of insulin resistance and is associated with increased risk of T2DM and cardiovascular events. We aimed to explore the TyG index and contrast-induced acute kidney injury (CI-AKI) in patients with type 2 diabetes who underwent coronary angiology.

**Methods:**

This study enrolled 928 patients with suspected coronary artery disease who underwent coronary angiology or percutaneous coronary intervention in Zhongda hospital. Patient data were divided into quartiles according to the TyG index: group 1: TyG ≤ 8.62; group 2: 8.62<TyG ≤ 9.04; group 3: 9.04<TyG ≤ 9.45; and group 4: TyG>9.45. CI-AKI was diagnosed according to the KIDIGO criteria. Demographic data, hematological parameters, coronary angiology data, and medications were all recorded. We calculated the TyG index using the following formula: ln [fasting TG (mg/dL)×FPG (mg/dL)/2].

**Results:**

Patients who developed CI-AKI exhibited significantly higher TyG index levels compared to patients who did not develop CI-AKI. The incidence of CI-AKI sharply increased with increasing TyG. Univariate and multivariate analysis identified TyG as an independent risk factor for CI-AKI. The AUC of the ROC curve was as high as 0.728 when the value of TyG was 8.88. The corresponding sensitivity was as high as 94.9%. Adding the variable TyG to the model for predicting CI-AKI risk further increased the predictive value of the model from 80.4% to 82%.

**Conclusions:**

High TyG is closely associated with increased incidence of CI-AKI, demonstrating that TyG is an independent risk factor for CI-AKI. TyG has potentially predictive value for CI-AKI and may play a crucial role in risk stratification in clinical practice.

## Introduction

Contrast-induced acute kidney injury (CI-AKI) refers to acute kidney complications after intravenous administration of contrast agents during angiography, such as those used for enhanced CT, enhanced MRI, or vascular interventional treatment (1, 2). The new diagnostic criterion proposed by the “Kidney Disease: Improving Global Outcomes (KDIGO)” research team indicates postoperative increased serum creatinine (Scr) by ≥26.5 μmol/l (0.3 mg/dl) or by at least 50% compared to preoperative Scr (3). Despite increased awareness of CI-AKI prevention over the past decade, the incidence of CI-AKI remains relatively high. Amin (4) reported that, in 2008, the incidence of AKI, using the KDIGO definition, was 19.7% of 31,532 patients with acute myocardial infarction (AMI). The National Cardiovascular Data Registry Cath-PCI registry enrolled 985,737 patients who underwent percutaneous coronary intervention (PCI) between June 2009 and June 2011 and reported that 7.1% of these patients experienced CI-AKI with 3,005 (0.3%) requiring new dialysis (5). CI-AKI is the third leading cause of hospital-associated acute kidney injury after low perfusion-related kidney injury and drug-induced kidney injury (6). There is no effective treatment for CI-AKI because the exact pathogenesis of CI-AKI has not been fully elucidated. Therefore, screening patients with high risk factors and adopting prompt preventive strategies are very important for reducing the incidence of CI-AKI.

Diabetes mellitus (DM) is one of the primary risk factors for CI-AKI and is an essential factor in risk stratification (7). Insulin resistance (IR) is an important pathophysiological process and risk factor for T2DM (8). IR is closely related to the prevalence of chronic kidney dysfunction (9). Li et al. discovered that the incidence of CI-AKI in patients with insulin resistance was comparable to that of diabetic patients, and a logistic regression analysis showed that the insulin resistance index HOMA-IR was an independent risk factor for CI-AKI (OR = 1.39) (10). The emerging triglyceride-glucose (TyG) index demonstrated a close relationship with insulin resistance (11). As a new index for evaluating insulin sensitivity, TyG is highly correlated with the HOMA-IR assessment model and the euglycemic-hyperinsulinemic clamp gold standard experiment (12, 13). It has been reported that TyG is associated with many cardiovascular diseases, such as acute myocardial infarction (14), cardiovascular events (15), arterial stiffness (16), and coronary artery calcification (17). The TyG index is becoming a promising alternative indicator of insulin resistance due to its efficacy, simplicity and low cost. The purpose of this study was to investigate the predictive value of the TyG index for the occurrence of CI-AKI in patients with T2DM undergoing coronary angiography and to provide new insights for optimizing the CI-AKI risk assessment model.

## Materials and Methods

### Study Population

This was a prospective single-center cohort study recruiting 928 consecutive patients who underwent coronary angiology between October 2017 and October 2019 in Zhongda Hospital, which is affiliated with Southeast University. All patients were well informed and signed an informed consent form before enrollment. Patients over 18 years old with T2DM who had undergone coronary angiology due to suspected heart disease were enrolled. The exclusion criteria were as follows: 1. allergy to the contrast agent; 2. chronic renal insufficiency with eGFR<30 ml/min/1.73 m^2^; 3. completed enhanced computed tomography, magnetic resonance, or vascular angiography procedures within the previous 2 weeks; 4. suffered acute kidney insufficiency or had taken nephrotoxic drugs within the previous 2 weeks; 5. severe liver/kidney dysfunction or severe infectious disease; and 6. malignant tumor. Patients with incomplete clinical data were also excluded.

Twenty-five patients with renal insufficiency with eGFR<30 ml/min/1.73 m^2^ were excluded. Nine patients with a history of previous contrast use within previous 2 weeks were excluded. Eight patients with severe liver/kidney dysfunction or severe infectious disease were excluded. Six patients with malignant tumor were excluded, and 7 patients with incomplete data (n = 7) were excluded. Patients taking nephrotoxic drugs or who suffered AKI due to medications (n = 5) were also excluded from the study. After all the nonqualifying patients were excluded, 928 subjects were analyzed. There were no significant differences in the baseline characteristics between the excluded and included subjects (**Table 1**).

**Table 1:** Beseline charateristics of included and excluded subjects.

Variables	Included subjects (n = 958)	Excluded subjects (n = 60)	P value
Male (n, %)	658 (68.7%)	42 (70%)	0.435
Age (years)	68.22 ± 10.6	70.1 ± 10.52	0.127
SBP (mmHg)	136.25 ± 19.50	138.72 ± 23.37	0.367
DBP (mmHg)	76.03 ± 12.16	77.04 ± 14.37	0.193
BMI (kg/m^2^)	25.01 ± 3.74	25.41 ± 3.52	0.562
Hypertension (n, %)	741 (77.3%)	50 (83.3%)	0.280
Hypotension (n,%)	14 (2.1%)	2 (3.3%)	0.258
Anemia (n, %)	7 (0.7%)	1 (1.4%)	0.427

SBP, systolic blood pressure; DBP, diastolic blood pressure; BMI, body mass index.

### Groups

According to the TyG index levels, patient data were divided into 4 quartiles: group 1: TyG ≤ 8.62; group 2: 8.62≤TyG ≤ 9.04; group 3: 9.04≤TyG ≤ 9.45; and group 4: TyG≥9.45.

### Data Collection

Demographics, past history, hematological parameters, coronary angiography data, and medication history were all recorded. Blood samples were collected from the cubital vein after participants fasted for at least 10 h. Preoperative Scr levels were measured upon hospital admission before the coronary angiology and PCI. The postoperative Scr level was measured within 1 week and evaluated for determining the occurrence of CI-AKI. FPG was measured using the hexokinase method. Blood lipid index, including triglyceride (TG), total cholesterol (TC), high-density lipoprotein cholesterol (HDL-C), and low-density lipoprotein cholesterol (HDL-C) levels were quantitated using an automatic biochemistry analyzer (Hitachi 7150, Japan). TyG was calculated using the following formula: TyG=ln [fasting TG (mg/dL)×FPG (mg/dL)/2] (18).

### Definitions and Follow-Ups

T2DM was diagnosed as followed: self-reported T2DM previously diagnosed by a physician; FPG ≥7.0 mmol/L according to the American Diabetes Association’s standards of medical care (10); and currently following treatment regimen with glycemic drugs to control blood glucose.

The new 2018 diagnostic criterion for CI-AKI proposed by the “Kidney Disease: Improving Global Outcomes (KDIGO)” research team was used in the study: increased Scr level by ≥26.5 μmol/l (0.3 mg/dl) or by at least 50% compared to baseline values within one week after administration of the contrast agent (3).

According to current international guidelines, CKD is defined as decreased kidney function demonstrated by a glomerular filtration rate (GFR) < 60 ml/min/1.73 m^2^ or markers of kidney damage, or both, for a duration of at least 3 months, regardless of the underlying cause (19).

According to previous literature and guidelines (20), standard prophylactic hydration protocols were defined as intravenous 0.9% NaCl administered at 3–4 ml/kg/h 4 h before and 4 h after contrast administration.

The primary outcome definition in the present study was CI-AKI, and the definition of CI-AKI was based on the preoperative and postoperative serum creatinine levels, as measured within one week after administration of the contrast agent. Therefore, we conducted only in-hospital follow-ups with a median follow-up time of approximately 10 days.

### Statistical Analysis

We used SPSS 19.0 statistical software for data analysis. Numerical data are expressed as the means ± standard deviation and were compared using independent sample *t*-tests. Data indicating poor normality are expressed as interquartile ranges, and rank-sum tests were used for the analysis. Categorical variables are reported in frequencies and percentages and compared using the χ^2^ test. Univariate and multivariate regression analyses were used to assess the risk factors. The predictive value of TyG for CI-AKI was evaluated using the ROC curve. *P* < 0.05 was defined criterion of significance.

## Results

### Baseline Characteristics of the CI-AKI and Non-CI-AKI Groups

A total of 928 patients with T2DM who underwent coronary angiography or PCI were included in this study. The average age of the study population was 68.32 ± 8.95 years; 658 were male; and a total of 197 (21.2%) patients developed CI-AKI. We compared the baseline characteristics of the CI-AKI and non-CI-AKI groups and found that patients with CI-AKI were older, had higher systolic and diastolic blood pressure levels, lower hydration rates during the perioperative period, lower preoperative eGFR levels, higher FPG, and higher triglyceride, TC, and LDL-c levels compared to the patients who did not develop CI-AKI. More importantly, mean TyG in the CI-AKI group was 9.15, which was significantly higher than that of the control group (TyG=8.87, P<0.001) (**Table 2**).

**Table 2:** Baseline characteristics of the non-CI-AKI and CI-AKI groups.

Variables	CI-AKI group (n = 197)	Non-CI-AKI (n = 731)	P-value
Male (n, %)	137 (69.5%)	521 (71.2%)	0.655
Age (years)	68.98 ± 9.90	67.55 ± 8.62	0.023*
SBP (mmHg)	137.65 ± 19.50	133.31 ± 19.40	<0.001*
DBP (mmHg)	81.03 ± 17.16	74.13 ± 10.4	<0.001*
BMI (kg/m^2^)	24.47 ± 3.92	25.45 ± 3.69	0.123
Hypertension (n, %)	149 (75.6%)	592 (81.0%)	0.097
Hypotension (n, %)	4 (2.1%)	10 (1.3%)	0.498
Anemia (n, %)	2 (1.0%)	5 (0.6%)	0.633
Chronic kidney dysfunction (n, %)	4 (2.1%)	15 (1.9%)	0.985
Hydration (n, %)	107 (54.3%)	629 (86.0%)	<0.001*
Coronary angiology			
PCI (n, %)	140 (76.1%)	520 (71.7%)	0.161
Dose of contrast agent P_50_ (P_25_–P_75_)(ml)	76 (50–92)	69 (45–83)	0.287
Number of lesion vessels	1.70 ± 1.07	1.61 ± 0.89	0.283
Number of stents	0.99 ± 0.45	0.75 ± 0.48	0.069
Hematological index			
Preoperative eGFR P_50_ (P_25_–P_75_) (ml/min/1.73m^2^)	78.7 (74.5–82.9)	82.3 (62.6–87.4)	0.042*
FBG P_50_(P_25_–P_75_) (mmol/L)	7.32 (7.01–7.93)	6.57 (6.01–8.09)	0.002*
TG P_50_ (P_25_–P_75_) (mmol/L)	1.58 (1.06–2.13)	1.16 (1.03–1.33)	<0.001*
TC (mmol/L)	4.66 (3.70–5.63)	3.80 (3.17–4.49)	<0.001*
HDL-c P_50_ (P_25_–P_75_) (mmol/L)	1.07 (0.94–1.25)	1.17 (0.97–1.39)	0.075
LDL-c P_50_ (P_25_–P_75_) (mmol/L)	2.65 (1.65–3.62)	2.21 (1.66–2.72)	<0.001*
TyG	9.15 ± 0.77	8.87 ± 0.43	<0.001*
HbAlc (%)	7.49 ± 1.51	7.65 ± 1.55	0.508
Medications			
Oral hypoglycemic drugs (n, %)	180 (91.4%)	660 (90.3%)	0.645
Insulin (n, %)	62 (31.5%)	204 (27.9%)	0.326
Aspirin (n, %)	174 (88.3%)	676 (92.5%)	0.062
ACEI/ARB (n, %)	113 (57.4%)	437 (59.8%)	0.539
Clopidogrel (n, %)	62 (31.5%)	270 (28.7%)	0.453
β-blocker (n, %)	167 (84.8%)	617 (84.4%)	0.900
CCB (n, %)	99 (50.3%)	395 (54.0%)	0.345
Statin (n, %)	192 (97.5%)	690 (95.7%)	0.259

SBP, systolic blood pressure; DBP, diastolic blood pressure; BMI, body mass index; PCI, percutaneous coronary intervention; CKD, chronic kidney dysfunction; CI-AKI, contrast induced-acute kidney injury; FPG, fasting plasma glucose; HbA1c, glycosylated hemoglobin; TC, total cholesterol; LDL-C, low density lipoprotein cholesterol; TyG, triglyceride-glucose; eGFR, estimated glomerular filtration rate; ACEI, angiotensin-converting enzyme inhibitors; ARB, angiotensin II receptor blocker; CCB, calcium channel blocker. * represents P < 0.05.

### Baseline Characteristics of All Groups

**Figure 1:**
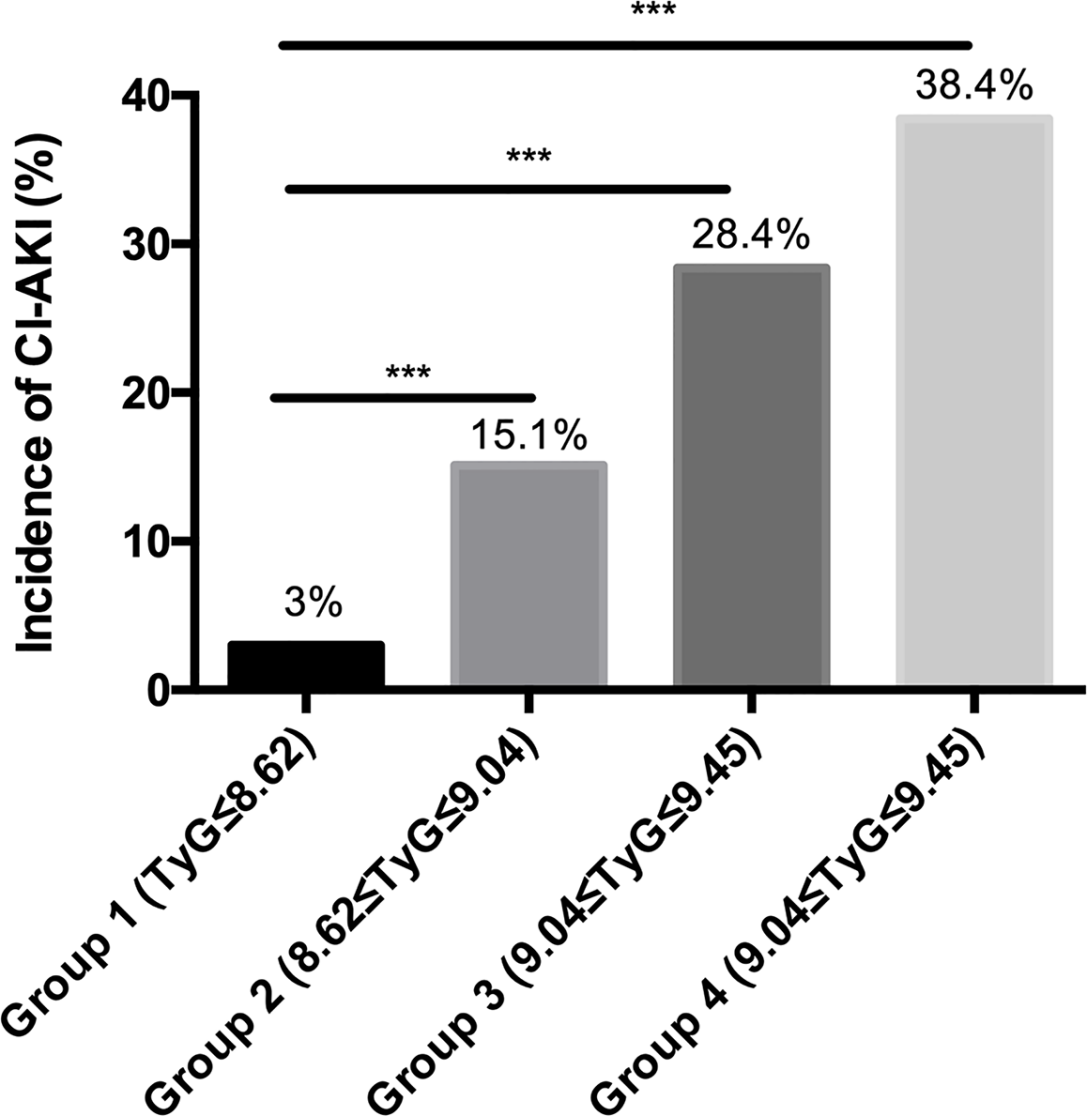
The incidence of CI-AKI in different TyG groups. *** represents P < 0.001.

Patient data were divided into quartiles according to the TyG index: group 1: TyG ≤ 8.62; group 2: 8.62<TyG ≤ 9.04; group 3: 9.04<TyG ≤ 9.45; and group 4: TyG>9.45. CI-AKI incidence significantly increased with increasing TyG, with only 3% of the patients in group 1 developing CI-AKI, while the incidence of CI-AKI in group 4 was as high as 38.4% (**Figure 1**). Both group 2 (15.1%) and group 3 (28.4%) exhibited significantly higher incidence of CI-AI (P<0.001). Patients in group 4 were younger with higher SBP, DBP, FPG, HbA1c, TG, TC, and LDL-c levels. The proportion of patients taking oral hypoglycemic drugs and CCB drugs were both higher in the groups with a high TyG index (**Table 3**).

**Table 3:** Baseline characteristics in four groups with different TyG levels.

Variables	Group 1 (n 232)	Group 2 (n = 232)	Group 3 (n = 232)	Group 4 (n = 232)	P-value
Baseline data					
Male (n, %)	170 (73.3%)	162 (69.8%)	169 (72.8%)	157 (67.7%)	0.501
Age (years)	70.60 ± 9.15	69.01 ± 8.79	70.80 ± 7.81	63.21 ± 7.50	<0.001*
SBP (mmHg)	135.34 ± 20.04	135.49 ± 17.99	136.86 ± 16.00	140.83 ± 17.60	<0.001*
DBP (mmHg)	74.99 ± 10.43	74.25 ± 9.54	75.47 ± 13.74	81.87 ± 12.59	<0.001*
BMI (kg/m^2^)	25.44 ± 3.17	24.47 ± 5.70	24.03 ± 5.34	24.13 ± 4.62	0.260
Hypertension (n, %)	178 (72.4%)	184 (79.3%)	177 (72.0%)	202 (85.8%)	0.013
Hypotension (n, %)	2 (0.9%)	3 (1.3%)	5 (2.2%)	4 (1.7%)	0.694
Anemia (n, %)	4 (1.7%)	2 (0.9%)	1 (0.4%)	0 (0.0%)	0.169
CKD (n, %)	8 (3.4%)	4 (1.7%)	6 (2.6%)	1 (0.4%)	0.124
Hydration (n, %)	187 (80.8%)	191 (82.6%)	180 (77.6%)	178 (76.7%)	0.409
CI-AKI	7 (3%)	35 (15.1%)	66 (28.4%)	89 (38.4%)	<0.001*
Coronary angiography					
PCI (n, %)	165 (69.4%)	161 (66.8%)	166 (71.6%)	178 (76.7%)	0.112
Dose of contrast agent P_50_ (P_25_–P_75_) (ml)	69 (45–83)	70 (50–92)	72 (50–92)	65 (50–92)	0.673
Number of lesion vessels	1.79 ± 0.64	1.65 ± 0.91	1.73 ± 0.54	1.52 ± 0.87	0.341
Number of stents	0.81 ± 0.34	1.02 ± 0.54	1.01 ± 0.48	0.92 ± 0.49	0.116
Hematological index					
Preoperative eGFR P_50_ (P_25_–P_75_) (ml/min/1.73m^2^)	87.6 (78.7–92.4)	85.7 (75.5–89.0)	84.7 (77.5–86.9)	86.5 (80.6–88.3)	0.329
FBG P_50_ (P_25_–P_75_) (mmol/L)	6.12 (4.94–6.35)	7.08 (5.90–8.33)	7.93 (6.79–9.00)	8.47 (7.19–12.06)	<0.001*
HbAlc (%)	7.60 ± 1.76	7.37 ± 1.46	7.47 ± 1.57	8.06 ± 1.60	<0.001*
Triglyceride P_50_ (P_25_–P_75_) (mmol/L)	0.69 (0.85–1.10)	1.16 (1.07–1.40)	1.68 (1.33–1.93)	2.74 (2.05–4.35)	<0.001*
TC P_50_ (P_25_–P_75_) (mmol/L)	3.42 (3.00–3.74)	3.91 (3.32–4.4)	4.05 (2.66–5.06)	4.65 (3.93–5.54)	<0.001*
HDL-c	1.18 (1.01–1.29)	1.11 (0.99–1.19)	1.09(0.98–1.13)	1.06 (0.93–1.10)	0.052
LDL-c	2.05 (1.46–2.26)	2.18 (1.83–2.77)	2.52 (1.37–3.06)	2.37 (1.72–3.28)	<0.001*
TyG index	8.28 ± 0.29	8.83 ± 0.13	9.22 ± 0.13	10.05 ± 0.53	<0.001*
Medications					
Oral hypoglycemic Drugs (n, %)	203 (87.5%)	205 (88.4%)	212 (91.4%)	220 (94.8%)	0.030*
Insulin (n, %)	61 (26.3%)	63 (27.2%)	68 (29.3%)	72 (31.0%)	0.667
Aspirin	207 (89.2%)	211 (90.9%)	217 (93.5%)	215 (92.7%)	0.347
ACEI/ARB	140 (60.3%)	130 (56.0%)	134 (57.8%)	145 (62.5%)	0.506
β-blocker	195 (84.1%)	192 (82.8%)	205 (87.2%)	192 (82.8%)	0.29
CCB	115 (49.6%)	114 (49.1%)	123 (53.0%)	142 (61.2%)	0.033*
Statin	222 (95.7%)	230 (99.1%)	229 (98.7%)	227 (97.8%)	0.051

SBP, systolic blood pressure; DBP, diastolic blood pressure; BMI, body mass index; CKD, chronic kidney dysfunction; CI-AKI, contrast induced-acute kidney injury; FPG, fasting plasma glucose; HbA1c, glycosylated hemoglobin; TC, total cholesterol; LDL-C, low density lipoprotein cholesterol; TyG, triglyceride-glucose; eGFR, estimated glomerular filtration rate; ACEI, angiotensin-converting enzyme inhibitors; ARB, angiotensin II receptor blocker; CCB, calcium channel blocker. * represents P < 0.05.

### The TyG Index Is a Risk Factor for CI-AKI After Coronary Angiology

Univariate and multivariate regression analyses were performed to identify independent risk factors for CI-AKI, and the results are presented in **Table 4**. Independent risk factors for CI-AKI include SBP (OR=1.025, 95% CI=1.012–1.038, P<0.001), hydration (OR=0.199, 95% CI=0.127–0.311, P<0.001), eGFR (OR=0.789, 95% CI=0.6298–0.869, P=0.024), LDL-c (OR=2.022, 95% CI=1.229–3.326, P=0.006), and the TyG index. Stratified analysis revealed that the incidence of CI-AKI was significantly increased in groups 2, 3, and 4 compared to that in group 1 (OR=1.431, 95% CI=1.170–2.170, P<0.001; OR=1.620, 95% CI=1.469–2.526, P<0.001; and OR=2.370, 95% CI=1.887–3.368, P<0.001, respectively).

**Table 4:** Univariate and multivariate analyses of patients with CI-AKI after coronary angiology.

Variables	Univariate analysis			Multivariate analysis		
	OR value	95% CI	P value	OR value	95% CI	P value
Sex (Male)	0.683	0.489–1.954	0.125	0.507	0.318–1.809	0.140
Age	1.025	1.013–1.058	0.023*	1.016	0.978–1.045	0.294
BMI	1.004	0.977–1.031	0.795	0.976	0.942–1.011	0.176
Hypertension	0.729	0.501–1.059	0.097	0.540	0.251–1.365	0.480
SBP	1.016	1.005–1.026	0.003*	1.025	1.012–1.038	<0.001*
DBP	1.024	1.008–1.040	0.003*	0.994	0.976–1.013	0.548
Hydration	0.193	0.136–0.274	<0.001*	0.199	0.127–0.311	<0.001*
eGFR	0.824	0.769–0.937	0.042*	0.789	0.628–0.869	0.024*
FBG	1.086	1.015–1.162	0.016*	0.982	0.868–1.112	0.780
HbA1c	0.931	0.838–1.035	0.188	0.805	0.694–1.133	0.121
Triglyceride	1.457	1.313–1.618	<0.001*	1.088	0.923–1.283	0.313
TC	1.928	1.652–2.249	<0.001*	0.746	0.483–1.154	0.189
HDL-c	0.836	0.682–1.037	0.075	0.946	0.806–1.025	0.53
LDL-c	1.427	1.223–1.666	<0.001*	2.022	1.229–3.326	0.006*
TyG group 1	1			1		
TyG group 2	1.711	1.481–2.145	<0.001*	1.431	1.170–2.170	<0.001*
TyG group 3	1.780	1.217–2.570	<0.001*	1.620	1.469–2.526	<0.001*
TyG group 4	2.005	1.012–3.407	<0.001*	2.370	1.887–3.368	<0.001*

SBP, systolic blood pressure; DBP, diastolic blood pressure; eGFR, estimated glomerular filtration rate; FPG, fasting plasma glucose; HbA1c, glycosylated hemoglobin; TC, total cholesterol; LDL-C, low density lipoprotein cholesterol; TyG, triglyceride-glucose. * represents P < 0.05.

**Figure 2:**
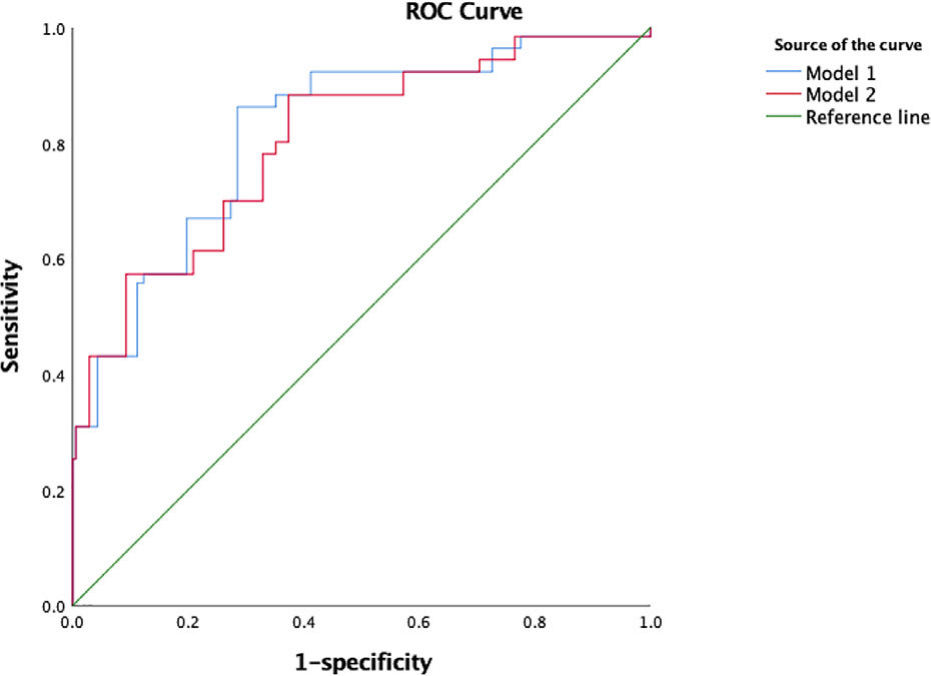
The ROC curve of model 1 and model 2 for predicting CI-AKI.

We combined the regression analysis results and previous research results on CI-AKI and included age, sex, hypertension, SBP, DBP, CKD, hydration, eGFR, FPG, TG, TC, and LDL-c levels in a new CI-AKI risk assessment model, and the results showed that the AUC for predicting CI-AKI in this model was as high as 0.804. The area under the ROC curve further increased to 0.820 after the TyG variable was added to the model (**Figure 2**), suggesting that TyG increases the diagnostic value of this risk prediction model (**Table 5**).

**Table 5:** ROC curve for TyG shows enhanced predictive value for CI-AKI.

Model	AUC	95% CI	P value
Model 1 (Model 2+TyG index)	0.820	0.787–0.854	0.017
Model 2	0.804	0.768–0.84	0.018

Model 2 includes age, sex, hypertension, SBP, DBP, CKD, hydration, eGFR, FPG, TG, TC, LDL-c.

**Figure 3:**
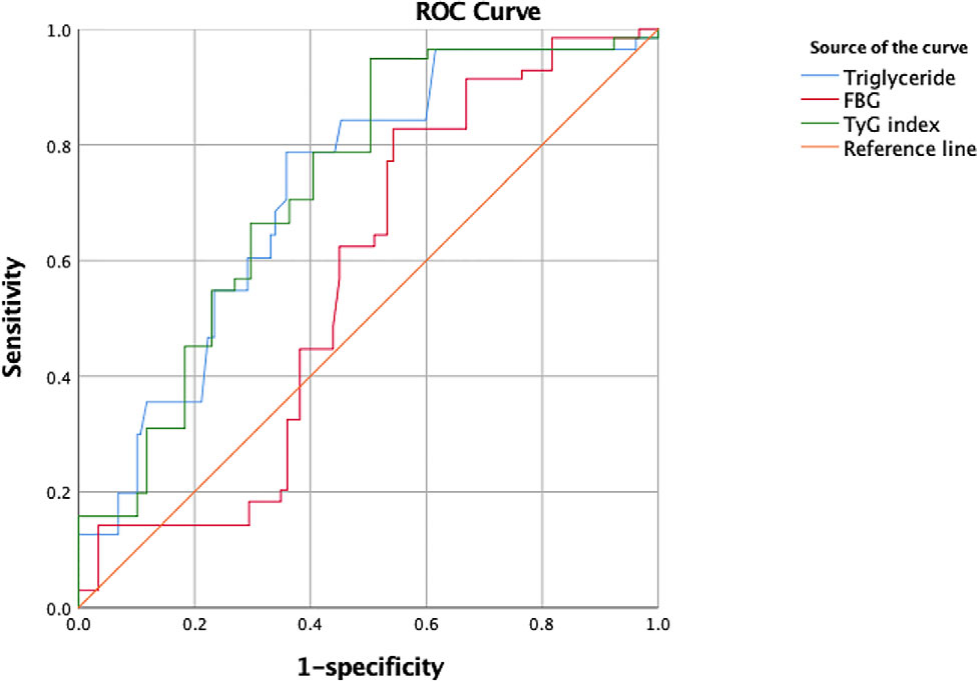
The ROC curve of triglyceride, FBG and TyG index for predicting CI-AKI.

We further compared the predictive value of the ROC curves for the FPG level, TyG index, and TG level to predict CI-AKI (**Figure 3**). The AUC of the TyG index for predicting CI-AKI in patients with T2DM who underwent angiology was 0.728 (95% CI=0.691–0.764, P=0.019) when the value of TyG was 8.88. The corresponding sensitivity was as high as 94.9%, and the specificity was 51.3%. The AUC of the FPG and TG levels for predicting CI-AKI was 0.722 (95% CI=0.684–0.760, P=0.019) and 0.570 (95% CI=0.530 0.610, P=0.020), respectively.

## Discussion

In this study, we investigated the predictive value of the TyG index for the occurrence of CI-AKI in T2DM patients after coronary angiography. Patients in groups 2, 3, and 4 exhibited a markedly increased incidence of CI-AKI compared to those in group 1. We demonstrated, for the first time, that TyG, a biomarker of insulin resistance, is an independent risk factor for CI-AKI and has good predictive value for the diagnosis of CI-AKI. The AUC of the TyG index predicting CI-AKI reached 0.728 (95% CI = 0.691–0.761, P = 0.019), and the corresponding sensitivity was 94.7%. We provide a risk assessment model, including age, sex, hypertension, SBP, DBP, CKD, hydration, eGFR, FBG, TG, TC, and LDL-c levels and the TyG index, the AUC of which was as high as 0.820. There were no significant differences in the AUC for conventional risk models 1 and 2 (with the TyG index added) (P=0.405). The reason that the AUC was not significantly different between the 2 models is because we included 2 variables, FBG and TG levels, in conventional model 1. The TyG index is calculated using the formula ln [fasting TG (mg/dL)×FPG (mg/dL)/2]. Therefore, it is understandable that the 2 models have similar predictive value for CI-AKI. Models 1 and 2 exhibited good predictive value, and both emphasized the significance of TyG index (FBG and TG) or insulin resistance. Furthermore, the slight difference might be distinguished in larger sample sizes. The AUC for the TyG index used for predicting CI-AKI was aberrantly higher than that for the TG level (0.570), while it was statistically similar with that for the FPG level. Both the TyG index and FPG level showed fine predictive value, but model 2, which combines additional independent risk factors with a larger AUC is recommended over a model based on a single variable.

CI-AKI is a serious complication of cardiac intervention and the third leading cause of acute kidney injury in the hospital, contributing to severe short-term and long-term adverse prognosis (21). CI-AKI is an incurable disease, but adequate intravenous hydration, avoiding the use of nephrotoxic drugs, choosing low osmotic or isotonic contrast agents and reducing the amount of contrast agents are all effective preventive measures to reduce the incidence of CI-AKI (22). Therefore, prompt risk assessment and timely prevention for high-risk patients are effective ways to reduce the incidence of CI-AKI.

In Tsai’s research, eGFR >60 ml/min/1.73 m^2^ was indicated to be normal if it was not in the setting of a unilateral kidney or in a patient with structural kidney disease (5). Referring to Tsai’s research and the CKD definition, the eGFR of the CKD patients in our research ranged from 30–60 ml/min/1.73 m^2^. There were a total of 19 CKD (11 stage 3A and 8 stage 3B) patients in our study. As shown in **Table 2**, no significant differences were found in the incidence of CKD between the 4 groups with different TyG levels, indicating preoperative eGFR level of the CKD patients did not influence the outcome in the current research. Furthermore, the incidence of CI-AKI increase was relatively flat between eGFR>60 ml/min/1.73 m^2^ and 30<eGFR<45 ml/min/1.73 m^2^ after coronary angiography with PCI, according to baseline serum creatinine (3). In conclusion, 19 (2.05%) patients with 30<eGFR<60 ml/min/1.73 m^2^ did not skew the research results.

Diabetes is a clear independent risk factor for CI-AKI. Diabetic kidneys are more susceptible to the effects of hypoxia and oxidative stress that aggravates the renal damage caused by contrast agents (23). Diabetic patients with CI-AKI have a worse prognosis compared to CI-AKI patients with normal blood glucose (24). Type 2 diabetes primarily occurs because of defects in insulin secretion and insulin resistance (25). Insulin resistance is a systemic disorder affecting several insulin-regulated pathways and many organs. It is characterized by reduced insulin effects, despite increased insulin concentrations in the blood. Insulin resistance is a hallmark of metabolic syndrome and a crucial characteristic for T2DM and increases the risk of cardiovascular events (26).

A series of epidemiological investigations demonstrated the role of insulin resistance in renal insufficiency, and insulin resistance plays a role in increasing vascular permeability (27). Insulin resistance is also associated with the occurrence of early glomerular hyperfiltration and contributes to late glomerular damage in the early stages of diabetic nephropathy (28, 29). Insulin signaling exerts crucial roles in renal hemodynamics, podocyte viability, and tubular function through the insulin receptor located on renal tubular cells and podocytes. Insulin resistance-associated hyperinsulinemia is related to metabolic syndrome, inflammation, and adipocytokine disorders, which can cause glomerular damage (9). The actions of insulin on renal sodium handling are preserved during hyperinsulinemia and contribute to increasing sodium reabsorption and increasing glomerular filtration rate, eventually leading to kidney damage (30). Glomerular endothelial cell injury, mesangial cell proliferation, and thickened basement membranes associated with insulin resistance related to oxidative stress ultimately cause glomerular sclerosis and renal tubular interstitial injury, leading to renal insufficiency (31).

As an emerging hematological indicator of insulin resistance, the products of triglycerides and fasting plasma glucose produced during fasting provide promising results that can be used to assess IR (13). TyG is a simple and convenient biomarker with high stability, and research has shown that the TyG index performs better than HOMA for the estimation of IR (12, 32). TyG has been shown to increase the risk of diabetes and various cardiovascular diseases. Low S and colleagues recruited 4,109 subjects without T2DM and discovered that patients in TyG quartiles 2, 3, and 4 exhibited significantly increased risk of developing T2DM compared to those in quartile 1 (33). Kahui Park et al. demonstrated in a study involving 1,175 Korean adults that the TyG index is associated with coronary artery calcification (CAC), and the TyG index is an independent risk factor for CAC (OR = 1.82, 95% CI=1.20-2.77, P<0.01) (17). An increased TyG index was significantly associated with an increased risk of developing cardiovascular disease and is thus useful for the early identification of CVD events in high-risk individuals (34).

Hyperglycemia also increases the risk of CI-AKI (35). Yin Wenjun et al. constructed a risk prediction model of CI-AKI with an AUC of 0.907 based on 8,800 patients who underwent angiography, and for the first time, preoperative blood glucose level was included in the predictive model (36). Barbieri L first demonstrated that elevated HbA1c is associated with an increased risk for CI-AKI among patients without prediabetes undergoing coronary angiography/PCI (37). However, in this study, the TyG index was independently correlated with CI-AKI, while neither HbA1c nor FPG was an independent risk factor for CI-AKI in the patients with T2DM who underwent coronary angiology. The TyG index is expected to become a novel predictive indicator for CI-AKI, providing new insight for CI-AKI risk stratification.

### Study Limitations

This study has the following limitations. First, this was a single-center study with a relatively small sample size. Second, changes in patient TyG index during hospitalization were not monitored. Therefore, multicenter studies with larger sample sizes are needed to further clarify the correlation between the TyG index and CI-AKI incidence.

## Conclusions

The current study demonstrates that a high TyG index is closely correlated with a higher incidence of CI-AKI in patients with T2DM undergoing coronary angiology, representing an independent risk factor for CI-AKI. The TyG index has enhanced predictive value, and furthermore, it attracted our attention because of its simplicity, convenience and low cost and because it might provide new insights into risk stratification in clinical practice.

## Data Availability Statement

The datasets generated for this study are available upon request sent to the corresponding author.

## Ethics Statement

The studies involving human participants were reviewed and approved by the Research Ethics Committee of the Affiliated Zhongda Hospital of Southeast University. The patients/participants provided their written informed consent to participate in this study.

## Author Contributions

CT, GY, and YQ contributed to the conception and design of the study. YHQ and HT collected the data, and YHQ, DW, YQ, EL, and JH participated in the data analysis and interpretation. YHQ wrote the manuscript with contributions from all authors. All authors contributed to the article and approved the submitted version.

## Funding

This work was supported by the National Natural Science Foundation of China (grant no. 81970237, 81600227).

## Conflict of Interest

The authors declare that the research was conducted in the absence of any commercial or financial relationships that could be construed as a potential conflict of interest.

## Abbreviations

AUC, area under the curve; CAC, coronary artery calcification; CI, confidence interval; CI-AKI, contrast-induced acute kidney injury; CVD, cardiovascular disease; eGFR, estimated glomerular filtration rate; FPG, fasting plasma glucose; HDL-C, high-density lipoprotein cholesterol; IR, insulin resistance; LDL-C, low-density lipoprotein cholesterol; OR, odd ratio; PCI, percutaneous coronary intervention; T2DM, type 2 diabetes mellitus; TC, total cholesterol; TG, triglyceride; and TyG, triglyceride-glucose.
